# Distinguishing between metastatic and benign adrenal masses in patients with extra-adrenal malignancies

**DOI:** 10.3389/fendo.2022.978730

**Published:** 2022-09-23

**Authors:** Jinchao Chen, Yedie He, Xiaowei Zeng, Shaoxing Zhu, Fangyin Li

**Affiliations:** Department of Urologic Surgery, The Cancer Hospital of the University of Chinese Academy of Sciences, Zhejiang Cancer Hospital, Institute of Basic Medicine and Cancer (IBMC), Chinese Academy of Sciences, Hangzhou, China

**Keywords:** adrenal, metastasis, differentiation, imaging, biopsy

## Abstract

**Background and Objectives:**

The adrenal gland is a common organ involved in metastasis. This study aimed to compare adrenal metastases (AMs) and adrenal benign masses (ABMs) of patients with extra-adrenal malignancies during the staging or follow-up.

**Methods:**

We retrospectively collected data from 120 patients with AMs and 87 patients with ABMs. The clinical characteristics, imaging features, pathology, and treatment regimes were analyzed.

**Results:**

The most common types of extra-adrenal malignancies in patients with ABMs included thyroid, kidney, and gynecological cancers. On the other hand, lung and kidney cancers and lymphoma were the most frequent primary cancers of AMs. The age and incidence of symptoms were significantly higher in patients with AM. Radiological analysis showed that AMs tended to have larger tumor sizes and higher attenuation values than ABMs on pre-contrast computed tomography (CT). The diagnostic accuracy of positron emission tomography-CT for AM was 94.1%. An adrenal biopsy had a diagnostic accuracy of 92.5%. A multivariate logistic regression model demonstrated that the origins of extra-adrenal malignancies, the enhancement pattern, and attenuation values in pre-contrast CT were independent predictors of AMs. The sensitivity and specificity of this predictive model of combination was 92.5% and 74.1%, respectively.

**Conclusions:**

The differential diagnosis between AMs and ABMs is extremely important. The combination of origin of first malignancy, enhancement pattern and CT value in non-enhanced phase is a valuable model for predicting AMs.

## Introduction

The adrenal gland is a common site for metastases. Adrenal metastases (AMs) were found in 26-36% of patients with a known carcinoma (1). Cancer cells mainly metastasis to the adrenal glands through hematogenous route. Adrenal metastasis usually indicates a poor prognosis. The median survival time of lung cancer patients with adrenal metastasis was only 5 months (2). Almost all solid malignancies may develop AM in the natural history of the disease, and the median interval for AMs is 2.5 years (3). AMs are generally asymptomatic and are often detected radiologically during staging or the follow-up of patients with extra-adrenal malignancies (4, 5). Adrenal masses found in patients with extra-adrenal malignancies should always be suspected of being metastases.

Adrenal benign masses (ABMs) are also not uncommon in patients with extra-adrenal malignancies. A previous study found that ABMs were found in 74% of patients who had a history of extra-adrenal malignancy (6). The accurate staging of malignancies is the basis of the optimal choice of treatment. Therefore, distinguishing AMs from ABMs during staging or follow-up is extremely important for patients with extra-adrenal malignancies. Previous studies have shown that the imaging features of AMs lack specificity due to the complexity of different primary malignancies (5). An adrenal mass biopsy is an important method to confirm the diagnosis, but the negative predictive value of biopsy for adrenal incidentalomas is low and the positive rate in patients with extra-adrenal malignancies is only 70% (7). Thus, AMs cannot be completely excluded based on biopsies and a more comprehensive comparison system is required to differentiate ABMs from AMs.

We systematically compared the clinical features, diagnosis, pathology, and treatments in patients with AMs or ABMs over the same period. Here, we provide a comprehensive overview of this challenging clinical scenario. Furthermore, we recommend an algorithm for evaluation of an adrenal mass in patient with extra-adrenal malignancy.

## Materials and methods

This study was reviewed and approved by the Medical Ethics Committee of Zhejiang Cancer Hospital. The database records of patients diagnosed with AM at Zhejiang Cancer Hospital from January 2008 to June 2021 were reviewed. Patients were included if: i) they had a pathologically confirmed malignancies in a location other than the adrenal gland, ii) they had AM that was pathologically diagnosed by biopsy or surgical resection, and iii) the source of the AM was consistent with the primary lesions. Patients were excluded if they had a direct extension of the tumor to the adrenal gland, instead of a true metastasis.

Data for patients with extra-adrenal malignancies who were diagnosed with ABMs during a staging workup or at follow-up were collected from January 2008 to June 2021. The diagnosis of ABM, including adenoma, pheochromocytoma, and myelolipoma, was confirmed by pathology. Though approximately 10 percent of pheochromocytomas are malignant, there has been no reliable method available to predict the malignant potential of pheochromocytoma. During the median follow-up of 62 months, no recurrence or metastasis was found in these patients with pheochromocytomas. Therefore, in this article, we classified pheochromocytomas as ABMs. Endocrine function (plasma catecholamines, urinary metanephrines, 24-hour urine-free cortisol, plasma aldosterone) was evaluated in all patients, and patients with positive results were excluded.

We totally retrospectively collected data from 120 patients with AM and 87 patients with ABM. The clinical data for these patients, including demographic data, treatment of extra-adrenal malignancies, symptoms, imaging features, pathology results, synchronous or metachronous adrenal mass based on the time of the extra‐adrenal malignancy diagnosis and treatment of adrenal masses, were analyzed. The location, size, margin, Hounsfield unit (HU) value, and enhancement patterns of adrenal lesions on computerized tomography (CT) and chemical shift magnetic resonance imaging (MRI) were evaluated by a radiologist. Adrenal adenomas are diagnosed by CT if they appear to be small and round or oval, with well-defined margins, and the pre-contrast CT attenuations are less than 10 HU (8, 9). AMs are diagnosed by CT if they are larger than 3 cm in diameter, and have a pre-contrast CT value > 30 HU and heterogeneous enhancement. The absolute percentage washout (APW) and relative percentage washout (RPW) were calculated as follows: (a) APW = (CT_E_ - CT_D_)*100/(CT_E_ - CT_U_) and (b) RPW = (CT_E_ - CT_D_)*100/CT_E_, where CT_U_, CT_E_, and CT_D_ represented the lesion attenuation values at unenhanced CT, early enhanced CT, and delayed enhanced CT, respectively (10). Adrenal uptake exceeding liver uptake or maximum standardized uptake value (SUVmax) > 2.5 was considered positron emission tomography (PET) positive (PET+) for metastasis. Biopsy and surgical specimens were evaluated by pathologists and, if necessary, immunohistochemical staining was performed to confirm the diagnosis.

Categorical and continuous variables were analyzed using the chi-square test and Student’s t-test, respectively. Multivariate logistic regression was used to identify the predictive factors for AM. All statistical analyses were performed using SPSS Statistics for Windows, version 19 (IBM Corp., Armonk, NY, USA). Statistical significance was set at *p* < 0.05.

## Results

### Origin, staging, and primary treatment of extra-adrenal malignancies

The extra-adrenal malignancies in patients with AM included lung (42.5%), renal (10.8%), colorectal (5.8%), liver (5.8%) and gynecological cancers (5.8%) and lymphoma (10%) (**Table 1**). The frequent extra-adrenal malignancies in patients with ABM comprised of thyroid (21.8%), kidney (14.9%), gynecological (13.8%), breast (12.6%), stomach (11.5%), lung (9.2%), and colorectal (5.7%) cancers. A higher percentage of patients with stage III/IV extra-adrenal tumors were found in the metastatic group and a higher percentage of those with stage I-II tumors were found in the benign group (*p* < 0.001).

**Table 1:** The origin of first primary cancers in patients with adrenal metastases and those with benign adrenal masses.

Origins	Metastatic	Benign	P-value
Total	120	87	
Lung	51 (42.5%)	8 (9.2%)	**<0.001**
Colorectal	7 (5.8%)	5 (5.7%)	
Breast	3 (2.5%)	11 (12.6%)	
Stomach	3 (2.5%)	10 (11.5%)	
Thyroid	1 (0.8%)	19 (21.8%)	
Nasopharynx	0 (0%)	2 (2.3%)	
Esophagus	2 (1.7%)	2 (2.3%)	
Gynecologic	7 (5.8%)	12 (13.8%)	
Hematologic	12 (10%)	0 (0%)	
Urinary tract	2 (1.7%)	3 (3.4%)	
Kidney	13 (10.8%)	13 (14.9%)	
Thymus	0 (0%)	1 (1.1%)	
Melanoma	2 (1.7%)	3 (3.4%)	
Liver	7 (5.8%)	0 (0%)	
Prostate	0 (0%)	1 (1.1%)	
Mesothelioma	1 (0.8%)	1 (1.1%)	
Bile duct	1 (0.8%)	0 (0%)	
Pancreas	3 (2.5%)	0 (0%)	
Unknown	5 (4.2%)	0 (0%)	

The bold values mean statistical significant.

### AM and ABM patients’ characteristics

The demographic details of the metastatic and benign groups are presented in **Table 2**. Patients with AM were older at diagnosis (59.9 *vs*. 55.4 years, *p* = 0.002) and had a higher male:female ratio (69.2% *vs*. 42.5%, *p* < 0.001) than patients with ABM. Metastases to other parts of body were more common in patients with AM (55.8% *vs*. 21.8%, *p* < 0.001). None of the patients with ABM had symptoms. However, 17.5% of patients with AM had clinical symptoms which consisted of abdominal pain (n=11, 9.2%), low back pain (n=8, 6.7%), intestinal obstruction (n=1, 0.83%) and obstructive jaundice (n=1, 0.83%).

**Table 2:** Demographics and clinical characteristics of patients with adrenal metastases and those with of benign adrenal masses.

Characteristic			Metastatic	Benign	P-value
Patients					
No.			120	87	
Gender		Male	83 (69.2%)	37 (42.5%)	**<0.001**
		Female	37 (30.8%)	50 (57.5%)	
Age (mean, year)			59.9 (32-80)	55.4 (21-77)	**0.002**
Stage of first primary cancers	I/II		17 (14.2%)	58 (66.7%)	**<0.001**
	III/IV		89 (74.2%)	17 (19.5%)
	Missing		14 (11.7%)	12 (13.8%)
Other site metastasis	Yes		67 (55.8%)	19 (21.8%)	**<0.001**
	No		41 (34.2%)	68 (78.2%)
	Unknown		12 (10%)	0
Adrenal masses					
Symptom	Yes		21 (17.5%)	0	**<0.001**
	No		99 (82.5%)	87 (100%)
Interval between first cancer to adrenal mass, month (range)			17.9 (0-205)	15.4 (0-312)	0.64
Synchronous			64 (53.3%)	62 (71.3%)	**0.009**
Metachronous			56 (46.7%)	25 (28.7%)
Laterality		Unilateral	89 (74.2%)	84 (96.6%)	**<0.001**
		Bilateral	31 (25.8%)	3 (3.4%)
Side		Left	52 (58.4%)	44 (52.4%)	0.424
		Right	37 (41.6%)	40 (47.6%)
Mean diameter of adrenal mass, cm (range)			4.6 (1.1-11.5)	2.6 (0.5-10.5)	**<0.001**
Mean linear growth rate, cm/month (range)			0.75 (0.39-1.3)	0.03 (0-0.15)	**<0.001**
Adrenal biopsy	Yes		67 (55.8%)	4 (4.6%)	**<0.001**
	No		53 (44.2%)	83 (95.4%)
Treatment	Adrenalectomy		57 (47.5%)	83 (95.4%)	**<0.001**
	Radiotherapy		6 (5%)	0
	Ablation		1 (0.8%)	0
	No local treatment		56 (46.7%)	4 (4.6%)

The bold values mean statistical significant.

### Features of metastatic and benign adrenal masses

The features of metastatic and benign adrenal masses are listed in **Table 2**. Synchronous adrenal masses were detected more frequently in the ABM group than in the AM group (71.3% *vs*. 53.3%, *p* = 0.009). Bilateral metastases were more common in patients with AM (25.8% *vs*. 3.4%, *p* < 0.001). The mean diameter of the AMs was 4.6 cm (1.1–11.5 cm), while the mean diameter of ABMs was 2.6 cm (0.5–10.5 cm, *P* < 0.001). However, there was no significant difference in time interval between the diagnosis of extra-adrenal malignancy and the detection of an adrenal mass between the two groups.

All patients underwent enhanced CT scans, and the diagnostic accuracy was 70% and 67.8% for the AM and ABM groups, respectively (**Table 3**). The enhanced CT imaging features showed that more AMs had irregular margins and heterogeneous enhancement patterns (*p* < 0.001). The mean pre-contrast attenuation value of the adrenal masses was significantly higher in the AM group than that in the ABM group (32.8 ± 5.9 *vs*. 15.3 ± 15.2, *p* < 0.001). However, there was no significant difference in the post-contrast attenuation value, APW and RPW of the adrenal masses between the two groups. The mean growth rate of the AMs was 0.75 cm/month with the median follow-up of 3.5 months and the mean growth rate of the ABMs was 0.03 cm/month with the median follow-up of 22.5 months (*p* < 0.001).

**Table 3:** Radiological characteristics of patients with adrenal metastases and those with benign adrenal masses.

Radiological evaluation		Metastatic	Benign	P
CT				
Radiological diagnosis	Benign	7 (5.8%)	59 (67.8%)	**<0.001**
	Metastasis	84 (70%)	10 (11.5%)	
	Unsure	9 (7.5%)	14 (16.1%)	
	Unknown	20 (16.7%)	4 (4.6%)	
Internal texture of adrenal mass	Homogeneous	78 (62%)	54 (65.1%)	0.659
	Heterogeneous	22 (38%)	29 (34.9%)	
Margin of adrenal mass	Smooth	78 (68%)	83 (100%)	**<0.001**
	Irregular	22 (32%)	0	
Attenuation values in non-enhanced phase (HU)		32.8 ± 5.9	15.3 ± 15.2	**<0.001**
Attenuation values in corticomedullary phase (HU)		48.4 ± 15.9	42.8 ± 23.8	0.122
Enhancement (HU)		18.7 ± 18.2	27.2 ± 19.1	**0.010**
Enhancement pattern	Homogeneous	26 (26%)	47 (56.6%)	**<0.001**
	Heterogeneous	72 (72%)	34 (41.0%)	
	No Enhancement	2 (2%)	2 (2.4%)	
APW		-149.1 ± 567.9	-165.0 ± 790.5	0.897
RPW		-18.2 ± 20.8	-29.3 ± 49.3	0.095
MRI				
Diagnostic accuracy		18/24 (75%)	18/20 (90%)	0.259
Hypo-intense on T1		22/24 (91.7%)	15/20 (75%)	0.217
Hyper-intense on T2		18/24 (75%)	18/20 (90%)	0.259
Signal suppression on chemical shift MRI		0	14/20 (70%)	**<0.001**
PET-CT diagnosis	Benign	2 (5.9%)	4 (57.1%)	**<0.001**
	Metastasis	32 (94.1%)	3 (42.9%)	

CT, computed tomography; MRI, magnetic resonance imaging; HU, Hounsfield unit; PET-CT, positron emission tomography-computerized tomography; APW, absolute percentage washout; RPW, relative percentage washout. The bold values mean statistical significant.

Twenty-four patients with AM and 20 patients with ABM received MRI with an accurate rate of 75% and 90%, respectively. On chemical shift MRI, 70% of the adrenal masses of ABM demonstrate signal suppression, while no patients with AM showed signal suppression (p<0.001) (**Table 3**). PET/CT using ^18^F-fluorodeoxyglucose (^18^F-FDG) was performed in 34 patients with AM, and the diagnostic accuracy was 94.1%. On the other hand, only seven patients with ABM underwent PET/CT, with a diagnostic accuracy of 57.1% (*p* < 0.001) (**Table 3**).

A total of 67 (55.8%) patients with AM underwent adrenal biopsy and 92.5% of these AMs were classified as metastatic lesions. Only four (4.6%) patients with ABM underwent adrenal biopsy (**Table 2**). There were no cases of needle metastasis or hemorrhage after the puncture.

Of the 120 patients with AM, 64 (53.3%) underwent local treatment, including adrenal metastasectomy (n = 57, 47.5%), radiotherapy (n = 6, 5%), and ablation (n = 1, 0.8%) (**Table 2**). The postoperative pathology results of the patients with AM confirmed the diagnosis of metastasis. There were 83 (95.4%) patients with ABM who underwent adrenalectomy. The postoperative pathology results of the patients with ABM showed adenoma (n = 72, 86.7%), pheochromocytoma (n = 9, 10.8%), and myelolipoma (n = 2, 2.4%).

**Figure 1:**
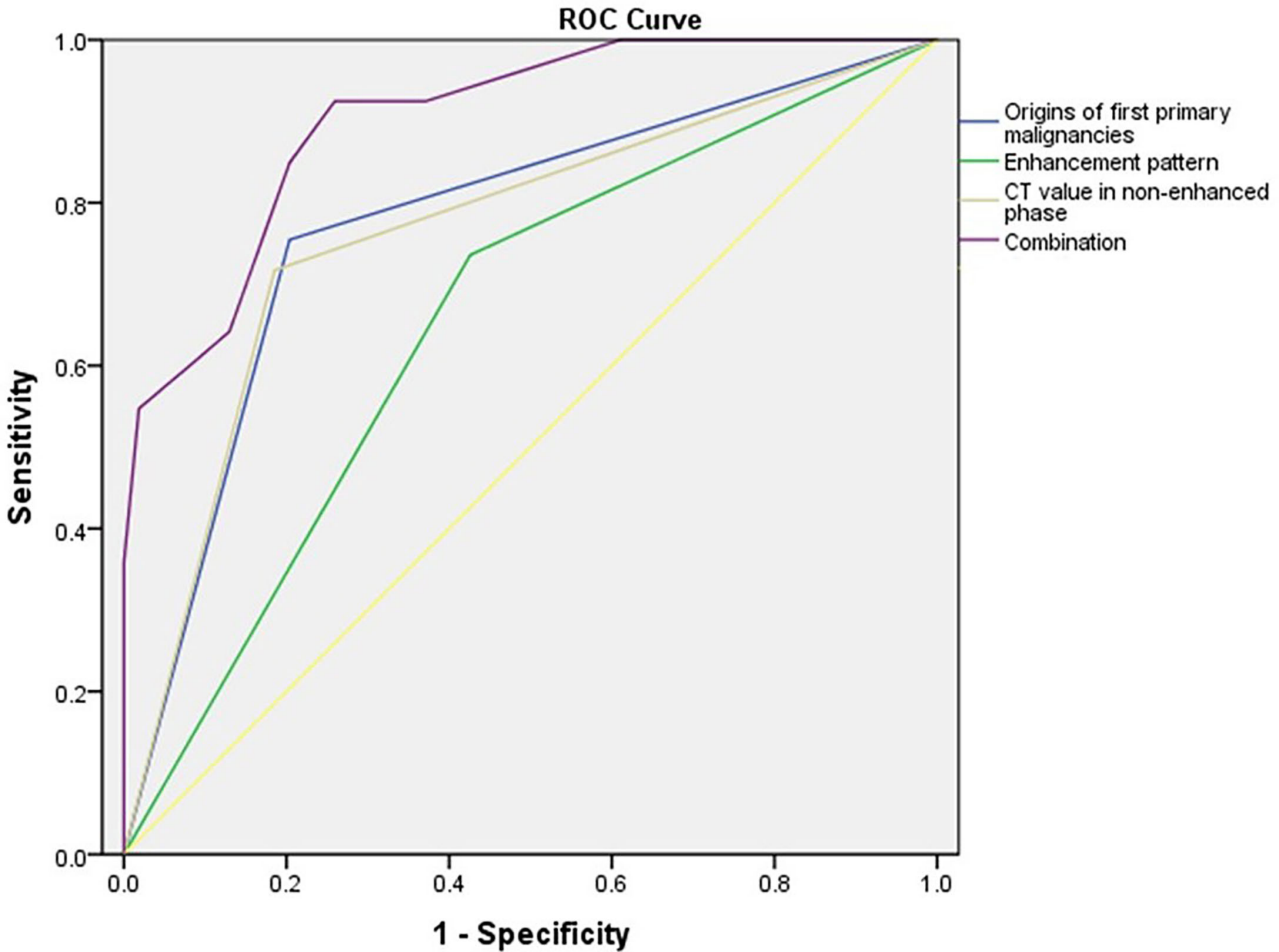
Receiver operating characteristic (ROC) curves for the differentiation of adrenal metastases and benign adrenal masses using a logistic regression model that included the origins of first primary malignancies, enhancement pattern, attenuation values in non-enhanced phase (HU) of adrenal mass and the combination. The area under the ROC curve (AUC) for each model is 0.776, 0.655, 0.766 and 0.902, respectively.

A multivariate logistic regression model revealed that the origins of the extra-adrenal malignancies, the enhancement pattern of the adrenal mass, and the adrenal mass pre-contrast HU value were predictive factors for metastasis (**Table 4**). Adrenal masses in patients with a history of lung or kidney cancer, a heterogeneous enhancement pattern, and a pre-contrast HU value ≥ 30 HU tended to be AMs. The area under the receiver operating characteristic (ROC) curve (AUC) for the model of combination of the origins of first primary malignancies, attenuation values in non-enhanced phase (HU) of adrenal mass and enhancement pattern reached 0.902 (**Figure 1**). The sensitivity and specificity of this predictive model of combination was 92.5% and 74.1%, respectively. The accuracy, positive predictive value and negative predictive value was 83.2%, 77.8% and 90.9%, respectively. When applying the predictive model of combination, 93 patients with AM and 79 patients with ABM could have been identified.

**Table 4:** Variables in the logistic regression diagnostic models to predict probability of metastasis for adrenal mass in patients with extra-adrenal malignancies.

Factors		HR	95% C.I.		P
Gender	Female	Reference			0.083
	Male	4.303	0.827	22.382	
Age		1.014	0.941	1.094	0.712
Origins of first primary malignancies	Others	Reference			**0.002**
	Lung and kidney	12.223	2.515	59.394
Stage of first primary malignancies	I-II	Reference			0.083
	III-IV	4.348	0.826	22.882
Other site metastasis	No	Reference			0.072
	Ys	5.111	0.863	30.258
Diameter of adrenal mass	<4cm	Reference			0.775
	≥4cm	1.308	0.208	8.230
Location of adrenal mass	Unilateral	Reference			0.169
	Bilateral	5.323	0.490	57.785
Enhancement pattern of adrenal mass	Homogeneous or no enhancement	Reference			**0.014**
	Heterogeneous enhancement	8.786	1.544	50.005
Attenuation values in non-enhanced phase (HU) of adrenal mass	<30	Reference			**0.001**
	≥30	15.021	2.934	76.898

HU, Hounsfield unit. The bold values mean statistical significant.

## Discussion

Both AMs and ABMs are common in patients with extra-adrenal malignancies. If an adrenal mass is detected radiologically at a cancer staging workup or a follow‐up consultation in a patient with an extra-adrenal malignancy, the differential diagnosis of AM or ABM is extremely important, because it determines the treatment plan. However, there have been few studies on this challenging clinical scenario (3, 6, 8, 11). In this study, the clinical manifestations, imaging features, pathology and treatment of AMs and ABMs were systematically compared to provide a reference for the effective differentiation of the two groups.

Our results showed a significant difference in the types of primary extra-adrenal malignancies between patients with AM and those with ABM. The common malignancies that metastasize to the adrenal gland include malignant melanoma, renal, breast, colon, lung, and bronchial carcinomas (3). Among these cancer types, lung cancer accounts for the highest proportion of adrenal gland metastases (39%), followed by breast cancer (35%), renal cell carcinoma (14%) and melanoma (12%) (3, 12). This is closely related to the high morbidity and biological characteristics of lung cancer (2, 3, 13). Our study found that lung cancer accounted for 42.5% of the primary lesions in patients with AM, which is consistent with the findings of previous studies (2, 3). For patients with ABM, the most common type of extra-adrenal malignancy was thyroid cancer (21.8%), which may be related to the high incidence of thyroid cancer in the study region. However, the large number of visits for thyroid diseases in our hospital may cause a certain selection bias. Differentiated thyroid carcinoma rarely metastasizes (14). Thyroid carcinoma accounted for only 0.8% of the primary lesions in patients with AM in this study. In terms of the staging of extra-adrenal malignancies, patients with AM were more likely to be in the late stage, while those with ABM were more likely to be in the early stage, which is consistent with the findings of previous studies (8, 15, 16).

We found that AM was more common in men. Byeon et al. also reported that the proportion of male patients with AM was much higher than the proportion with ABM (17). This may be due to that lung cancer accounts for the highest proportion of AM and are most likely seen among men (18). Moreover, thyroid cancer accounted for the highest proportion of extra-adrenal malignancies in the ABM group, and the incidence of thyroid cancer was significantly higher in women (13). Furthermore, there were more female patients with ABM (19). These factors may have contributed to the difference in the sex ratio between the two groups.

Adrenal incidentaloma (AI) increases with age (19). Radiological studies report a frequency of around 3% in the age of 50 years, and increases up to 10% in the elderly (20). It is rare in subjects below 40 years of age (19). In our study, the mean age of patients with ABMs was 55.4 years and ABMs were most commonly seen in patients ranged from 50 to 60 years. The patients with AMs were approximately four years older than those with ABMs in our study. This might be related to the different type of extra-adrenal malignancies between the two groups.

Size is an important parameter for distinguishing the nature of adrenal masses (5). For solitary adrenal masses larger than 3 cm in diameter in patients with known extra-adrenal malignancies, AMs should be suspected (5). Sasaguri et al. reported that an adrenal mass > 4 cm has a predictive value of 95.5% for metastasis (8). And a tumor diameter > 1.8 cm was found to be an independent predictor of AMs (21). Although our study confirmed that AMs were significantly larger than ABMs, a multivariate analysis showed that tumor diameter was not an independent predictor of AM, which was consistent with the results reported by Byeon et al. (17). For patients suspected of ABMs, if the size are more than 4 cm, resection of the adrenal masses were suggested (3). And for patients suspected of clinically isolated AMs regardless of size, resection were also suggested in our study. The bias of patient selection may affect the results of tumor size comparison between the two groups. Size change of adrenal masses is another factor to differentiate the nature (8, 22). Our study showed a significant higher growth rate of AMs than ABMs (0.75 *vs*. 0.03 cm/month). Sasaguri et al. reported a mean growth rate of 1 mm/year of ABMs and a mean growth rate of 29 mm/year of AMs (8). A rapid increase (>8 mm/year) of adrenal masses should be considered the potential of malignancy (22).

CT is a useful method for identifying the nature of adrenal masses (9). The value of the differential diagnosis of adrenal masses by CT mainly depends on the CT value, margins, and enhancement pattern (9). Adrenal adenomas are generally small and round or oval, with well-defined margins, and the pre-contrast CT attenuations are less than 10 HU, with a faster washout of contrast material on delayed contrast-enhanced CT images (8, 9). However, AMs are generally bilateral, oval or irregular, heterogeneous with ill-defined margins, and have a thick enhancing rim on contrast studies (8). Previous studies found that adrenal masses with a pre-contrast attenuation of > 43 HU or 36.2 HU should be suspected as being AMs, and had high sensitivity and specificity of diagnosis (17, 23). The sensitivity and specificity of an APW ≥ 60% in the diagnosis of adrenal adenomas were 88%–98% and 92%, respectively, and the sensitivity and specificity of an RPW ≥ 40% in the diagnosis of adrenal adenomas were 96% and 100%, respectively (9). In our study, AMs tended to be larger; bilateral; and have irregular margins, heterogeneous enhancement, and high pre-contrast CT values. Moreover, a pre-contrast CT value > 30 HU was an independent predictor of AMs. However, according to recent studies, CT has relatively high false positive and false negative rates for the diagnosis of AMs (24). In our study, the AUC for the model of combination of the origins of first primary malignancies, attenuation values in non-enhanced phase (HU) of adrenal mass and enhancement pattern reached 0.902. The sensitivity and specificity of this predictive model of combination was 92.5% and 74.1%, respectively. We believe that this may be a valuable predictive model for the diagnosis of AM. As a consequence, in some cases biopsy could be avoided.

MRI has been claimed to be somewhat more sensitive in finding lipid content in adrenal lesions than unenhanced CT (6). Adrenal adenomas with a high content of intracellular lipid usually lose signal intensity on out-of-phase images compared with in-phase images, whereas malignant lesions and pheochromocytomas that all lack intracellular lipid remain unchanged (20). Thus MRI has high accuracy for lipid-rich adenoma detection (95-98%) (19). Our study showed that the accuracy of MRI in the diagnosis of ABM is 90%, while the accuracy of MRI in the diagnosis of AM is 75%. However, recent guidelines suggest that MRI with chemical shift imaging should only be the first choice where CT is less desirable (19).

FDG-PET/CT has been shown to be highly sensitive and specific for the diagnosis of adrenal tumors (9). In a previous study, Boland et al. reported 100% sensitivity and specificity for PET in differentiating adrenal masses as benign or malignant in patients with known primary cancers (25). We found that PET/CT had a high accuracy of 94.1% for the diagnosis of AMs, but only 57.1% accuracy for the diagnosis of ABMs. Therefore, ABMs diagnosed by PET/CT still need to be closely monitored to further exclude the possibility of AMs.

Adrenal biopsy is the most important method for diagnosis of AMs (3). Several studies have shown that the sensitivity of fine needle aspiration (FNA) for the diagnosis of malignancy ranges from 81% to 100% and the specificity ranges from 83% to 100% (3). In patients with a confirmed extra-adrenal malignancy, percutaneous FNA has a positive predictive value of 100% and a negative predictive value of 92% (3). The results of a meta-analysis of 2,174 patients from 32 studies showed that the sensitivity and specificity of adrenal biopsy for the diagnosis of AMs were 87% and 96%, respectively (26). In our study, the diagnostic accuracy of adrenal biopsy in patients with AMs was 92.5%. However, as an invasive procedure, the complications of adrenal biopsy cannot be ignored. A meta-analysis showed that the overall complication rate of adrenal biopsy is 2.5% (26). The main complications include adrenal hematoma, pancreatitis, pneumothorax, hemothorax, perirenal hematoma, duodenal hematoma, and hypertension crisis (26). Therefore, the European Society of Endocrinology suggests performing a biopsy of an adrenal mass only if all of the following criteria are fulfilled: (i) the lesion is hormonally inactive, (ii) the lesion has not been conclusively characterized as benign by imaging and (iii) management would be altered by knowledge of the histology (20). Biopsy should never be performed prior to excluding the diagnosis of catecholamine-producing tumors because of the potentially life-threatening complications such as hypertensive crisis, stroke and myocardial infarction (26). In addition, adrenal biopsy is not recommended for patients who are not clinically considered to have AMs (20). In our study, few (4.6%) patients with a clinical diagnosis of ABM received adrenal biopsy, because the biopsy did not affect the treatment decision for these patients.

Adrenocortical carcinomas (ACCs) are rare, lethal malignancies with poor overall survival (27). It is sometimes quite difficult to distinguish adrenocortical carcinoma from AMs, for both of them are typically large, heterogeneous masses that do not demonstrate signal suppression on chemical shift imaging and often show areas of hemorrhage and necrosis (3). However, the probability of bilateral occurrence of adrenal metastases is higher than that of ACCs (4, 27). The patients with ACCs may have the history of symptoms of hormonal excess, and multiple adrenal hormones are elevated in about 26% to 94% of ACCs (3). When differentiating from AMs, other relatively rare type of benign adrenal masses should also be considered such as cavernous hemangioma, cysts, adenomatoid tumors and sex-cord stromal tumors (28).

The present study has several limitations. First, this study was retrospective and hence, recall errors may have occurred and affected the results. Second, this study excluded patients with adrenal masses only detected by imaging, without pathology results; thus, there may be selection bias. Third, in our study, we used 24h-urinary free cortisol instead of 1 mg dexamethasone suppression test as a screening test for ruling out Cushing syndrome, which may result in missing the diagnosis of autonomous cortisol secreting in some patients.

## Conclusion

**Figure 2:**
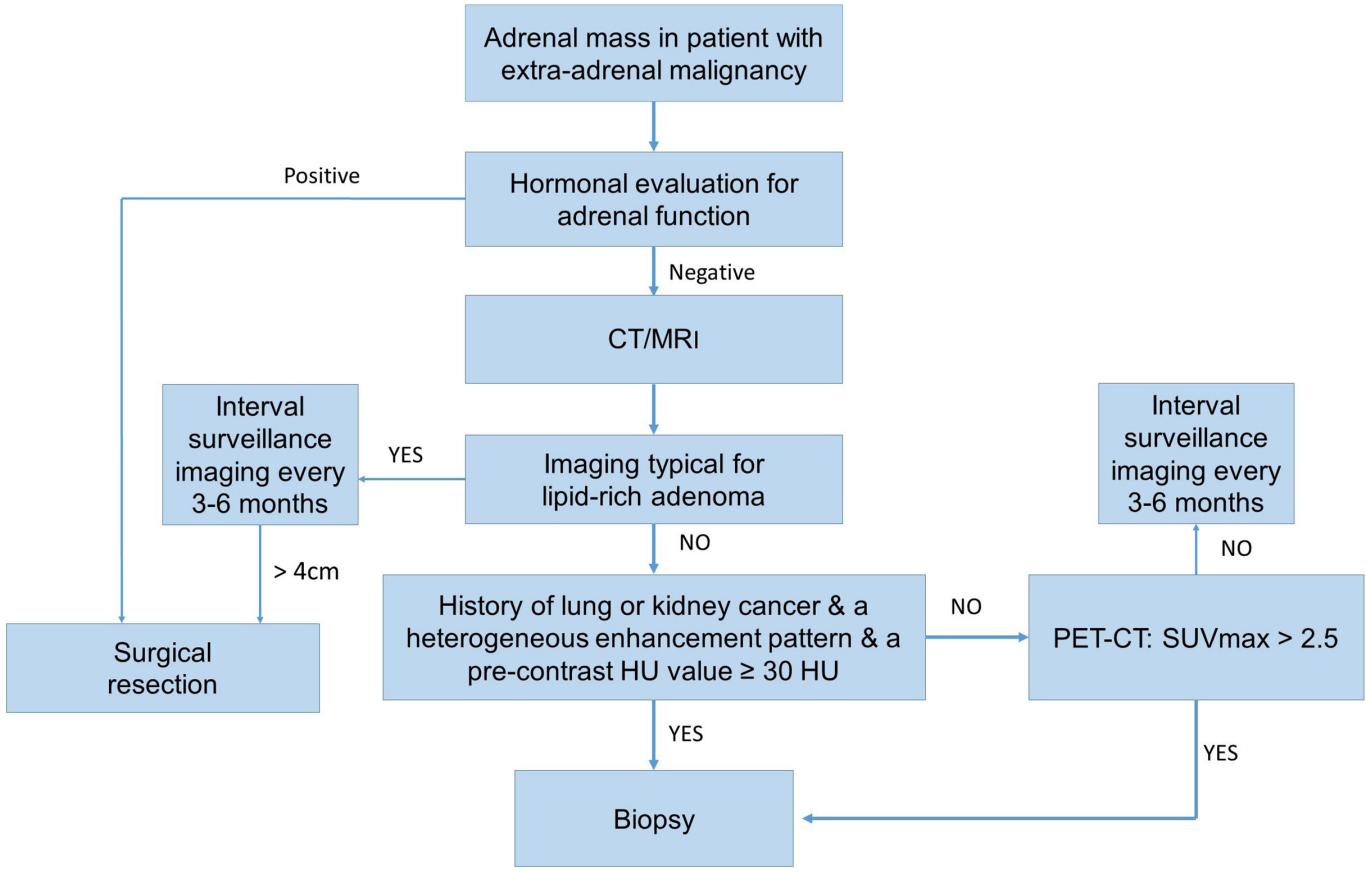
The operative flow-chart for evaluation of an adrenal mass in patient with extra-adrenal malignancy.

AMs are common in patients with extra-adrenal malignancies and they need to be differentiated from ABMs when making treatment decisions. AMs are frequently derived from lung and kidney cancers. On the other hand, patients with thyroid and kidney cancers are frequently associated with ABMs. Therefore, particularly in patients with kidney cancer it could be difficult to differentiate an AM from an ABM. AMs are characterized by a higher incidence of symptoms, a larger size, bilateral location, and extra-adrenal metastasis. CT is an important method for differentiating AMs from ABMs. PET/CT and adrenal biopsy are highly accurate for diagnosing of AMs. Adrenal masses in patients with a history of lung or kidney cancer, a heterogeneous enhancement pattern, and a pre-contrast HU value ≥ 30 HU tended to be AMs. We recommend the algorithm for evaluation of an adrenal mass in patient with extra-adrenal malignancy (**Figure 2**).

## Data availability statement

The raw data supporting the conclusions of this article will be made available by the authors, without undue reservation.

## Ethics statement

The studies involving human participants were reviewed and approved by the Medical Ethics Committee of Zhejiang Cancer Hospital. The patients/participants provided their written informed consent to participate in this study.

## Author contributions

SZ and FL was responsible for the concept and design of the study. JC, YH, and XZ dealt with the clinical data. JC performed the statistical work and drafted the manuscript. All authors contributed to the article and approved the submitted version.

## Funding

This study was supported by the Medical Health Science and Technology Project of Zhejiang Provincial Health Commission (Grant Number: 2021KY584).

## Conflict of interest

The authors declare that the research was conducted in the absence of any commercial or financial relationships that could be construed as a potential conflict of interest.

## Publisher’s note

All claims expressed in this article are solely those of the authors and do not necessarily represent those of their affiliated organizations, or those of the publisher, the editors and the reviewers. Any product that may be evaluated in this article, or claim that may be made by its manufacturer, is not guaranteed or endorsed by the publisher.
